# Multilocus Characterization Reveals an ITS-Defined Haplotype Associated with Pathogenic Variation in *Magnaporthe oryzae*

**DOI:** 10.3390/jof12070501

**Published:** 2026-07-09

**Authors:** Hafiz Muhammad Usman Aslam, Mark L. Gleason, Saba Aslam, Luqman Amrao

**Affiliations:** 1 Plant Pathology Program, San Luis Valley Research Center, Colorado State University, 249 E County Road 9 N, Center, CO 81125, USA; 2 Department of Plant Pathology, University of Agriculture, Faisalabad 38000, Pakistan; 2012ag2717@uaf.edu.pk (S.A.); raoluqman@gmail.com (L.A.); 3 Department of Plant Pathology, Entomology and Microbiology, Iowa State University, Ames, IA 50011, USA; mgleason@iastate.edu

**Keywords:** rice blast, disease severity, phylogenetics, *Oryza sativa*, fungal diversity

## Abstract

Rice blast, caused by *Magnaporthe oryzae*, is a major constraint to rice production worldwide. This study aimed to investigate the morphological, pathogenic, and molecular diversity of *M. oryzae* isolates collected from rice-growing regions of Punjab, Pakistan. A total of 125 infected samples were collected, from which 25 representative isolates were selected to represent the observed morphological diversity. Morphological characterization grouped the isolates into four distinct categories based on cultural and conidial characteristics. Pathogenicity assays under greenhouse conditions confirmed that all the tested isolates were pathogenic and fulfilled Koch’s postulates, with significant variation in disease severity and area under the disease progress curve (AUDPC). Isolate RBNN-1 produced the highest disease severity, whereas RBNR-3 produced the lowest. Multilocus molecular identification using ITS, β-tubulin, actin, and calmodulin gene regions confirmed all isolates as *M. oryzae*. Sequence analysis of the 25 representative isolates revealed high conservation across protein-coding genes, whereas a single nucleotide polymorphism (A→G) in the ITS region identified an ITS-defined haplotype present in seven isolates. Phylogenetic analysis placed these isolates in a well-supported subclade that was consistently associated with a distinct morphological group and higher disease severity under greenhouse conditions. These findings identified an ITS-defined haplotype that was consistently associated with a distinct morphological group and differences in disease severity among the analyzed isolates. This preliminary observation provides a foundation for future studies using higher-resolution genomic approaches to validate and further investigate intraspecific diversity in *M. oryzae*.

## 1. Introduction

Rice (*Oryza sativa* L.) is one of the most important cereal crops worldwide and plays a central role in global food security, providing a primary source of calories for more than half of the world’s population [1,2]. In Pakistan, rice is not only a staple food crop but also a major contributor to the national economy through domestic consumption and export earnings. Despite its importance, rice productivity in Pakistan remains relatively low compared to other major rice-producing countries, largely due to biotic stresses, particularly fungal diseases, and limited adoption of integrated disease management strategies.

Among the diseases affecting rice, blast caused by *M. oryzae* is considered the most destructive and widely distributed [3]. The pathogen exhibits a hemibiotrophic lifestyle and can infect rice plants at all growth stages, producing lesions on leaves, collars, nodes, necks, and panicles [4]. Neck and panicle blast are particularly damaging, as infection at these stages can lead to partial or complete sterility and severe yield losses [5,6]. Under favorable environmental conditions, rice blast epidemics can reduce yields by 30–70% or more in susceptible cultivars [4,7]. Globally, rice blast is responsible for substantial economic losses each year, highlighting its significance as a major constraint to rice production.

In Pakistan, rice blast has been reported frequently over the past two decades, particularly in Punjab Province, which is the country’s principal rice-growing region [8,9]. Districts such as Toba Tek Singh, Faisalabad, Gaggoo Mandi, and Vehari have experienced repeated outbreaks, reflecting the persistent nature of the disease. Climatic conditions characterized by high humidity, frequent rainfall, and moderate temperatures during the cropping season favor disease development and pathogen spread [10]. Current management strategies rely primarily on fungicide applications and the cultivation of resistant or moderately resistant varieties [9,11]. However, these approaches often provide only short-term control due to the pathogen’s ability to develop fungicide resistance and overcome host resistance.

The effectiveness and durability of rice blast management strategies are strongly influenced by the genetic diversity and evolutionary potential of *M. oryzae* populations. The pathogen is well known for its high genetic variability, rapid evolution, and ability to overcome host resistance genes [12,13]. The breakdown of resistance in previously resistant rice cultivars has been widely reported and is often associated with the emergence of new or genetically distinct pathogen variants [14,15]. Similarly, repeated use of fungicides with similar modes of action can lead to the development of resistant pathogen populations [16]. These challenges emphasize the need to understand pathogen diversity and population structure at regional and local scales.

Accurate identification and characterization of fungal pathogens are essential for understanding their diversity and epidemiology. Traditionally, *M. oryzae* identification has relied on morphological features such as cultural characteristics, conidial shape, size and septation. However, these traits can be influenced by environmental conditions and often lack sufficient resolution to distinguish closely related taxa or intraspecific variation, necessitating the use of molecular approaches [17,18]. Advances in molecular biology and DNA sequencing have significantly improved the accuracy of fungal identification and classification. Several genomic regions, including the internal transcribed spacer (ITS), β-tubulin, actin and calmodulin, have been widely used for the molecular identification and phylogenetic characterization of *M. oryzae* and related *Pyricularia* species, although the resolution provided by individual loci may vary depending on the study objectives [19,20,21,22].

The ITS region is commonly used as a universal DNA barcode for fungi due to its high copy number, ease of amplification, and ability to reveal genetic variation [20]. Although ITS alone may not resolve closely related taxa, it remains a valuable marker for detecting intraspecific variation. To overcome the limitations of single-locus approaches, multilocus sequence analysis has been increasingly adopted. By combining conserved and variable gene regions, multilocus approaches provide greater resolution for understanding evolutionary relationships, detecting genetic diversity, and assessing population structure [17].

Previous studies have employed a range of molecular approaches, including multilocus phylogenetic analysis, multilocus genealogy, comparative phylogenetic analysis, and RFLP analysis, to investigate the taxonomy, phylogenetic relationships, and genetic diversity of *M. oryzae* and related *Pyricularia* species [21,23,24,25]. Collectively, these studies have demonstrated the value of molecular markers for species identification and phylogenetic characterization of *Magnaporthe*/*Pyricularia* isolates. However, despite the economic importance of rice blast in Pakistan, studies integrating morphological characterization with multilocus molecular analysis and phylogenetic reconstruction of *M. oryzae* isolates remain limited. In particular, there is a lack of information on the extent of genetic variation within local populations and its relationship with disease severity.

Therefore, the present study was undertaken to investigate the morphological, pathogenic, and molecular diversity of *M. oryzae* isolates collected from major rice-growing areas of Punjab Province, Pakistan. The study integrates classical morphological characterization, pathogenicity assays, and multilocus molecular identification coupled with phylogenetic analysis to elucidate genetic relationships and variation in disease severity among the isolates.

## 2. Materials and Methods

### 2.1. Fungal Sampling and Isolation

**Figure 1:**
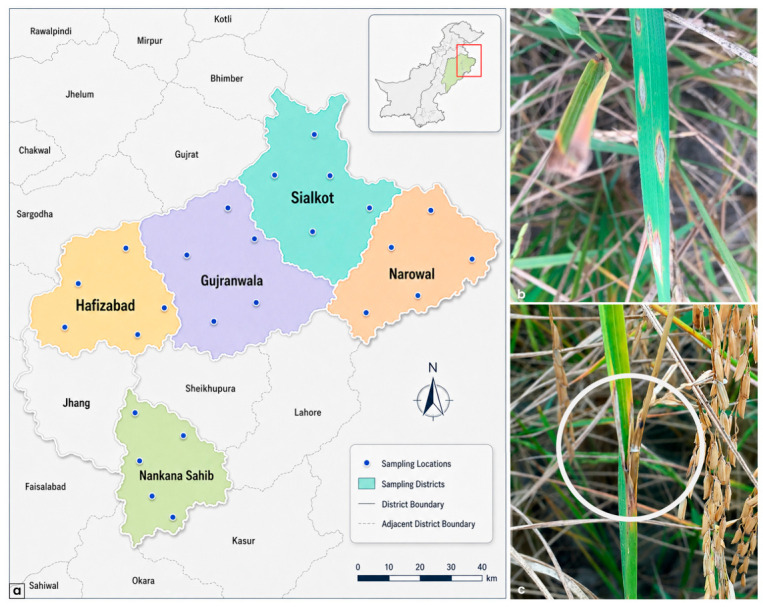
Sampling locations and representative rice blast symptoms observed in Punjab, Pakistan. (**a**) Map showing the five rice-growing districts surveyed (Nankana Sahib, Hafizabad, Gujranwala, Sialkot and Narowal) and the corresponding sampling villages (blue dots). The inset indicates the location of the study area within Pakistan. (**b**) Typical spindle-shaped leaf blast lesions caused by *M. oryzae*. (**c**) Typical neck blast symptoms observed on rice panicles, characterized by necrosis at the panicle neck leading to partial or complete panicle sterility.

A total of 125 rice blast-infected samples (leaf and panicle tissues) were initially collected from major rice-growing areas of Punjab Province, Pakistan, including Nankana Sahib, Narowal, Hafizabad, Sialkot, and Gujranwala, during the 2016–2018 cropping seasons. Within each district, representative villages were selected based on the occurrence of rice blast disease. In each village, five rice fields with a previous history of blast disease were selected following consultation with local farmers and samples were collected from plants exhibiting typical blast symptoms (Figure 1). The geographic coordinates of each sampling village were recorded using a handheld GPS device (Garmin Montana 680T, Garmin Ltd., Olathe, KS, USA). A sampling location map (Figure 1a) was generated in ArcGIS Pro 3.4 (Esri, Redlands, CA, USA) using the recorded village GPS coordinates and subsequently refined in Adobe Illustrator version 28.5 (Adobe Inc., San Jose, CA, USA). Detailed information on the sampling districts, villages, village GPS coordinates, number of fields sampled, host and plant tissues is provided in Supplementary Table S1.

For fungal isolation, small tissue segments (~5 mm^2^) were excised from the margins of actively expanding lesions, including a portion of adjacent healthy tissue. The segments were surface-sterilized in 1% sodium hypochlorite for 1 min, rinsed twice with sterile distilled water, blotted dry on sterile filter paper, and plated onto potato dextrose agar (PDA). Plates were incubated at 26 ± 2 °C under a 12-h photoperiod for 6–9 days to allow fungal growth. Emerging fungal growth were subcultured to obtain pure cultures [26]. Following isolation, all 125 isolates were subjected to morphological characterization. Based on the observed phenotypic diversity, 25 representative isolates (five isolates per district) were selected to represent the observed morphological variation and were subsequently subjected to independent pathogenicity, molecular identification and phylogenetic analysis.

### 2.2. Morphological Characterization

Twenty-five fungal isolates were morphologically characterized using a compound light microscope (Zeiss Axioscope, Oberkochen, Germany). Cultural characteristics, including growth pattern, pigmentation, and colony appearance, were recorded from cultures grown on PDA. Microscopic observations focused on conidial morphology (shape and septation) and hyphal characteristics (pigmentation and septation), which were assessed qualitatively. Conidial size (length × width) was measured and mean values were calculated based on measurements from 50 conidia per isolate. Based on the observed cultural and microscopic characteristics, including growth pattern, pigmentation, conidial shape, conidial size, and septation, the isolates were grouped into four distinct morphological categories.

### 2.3. Pathogenicity Assay and Statistical Analysis

Pathogenicity assays were conducted to confirm the pathogenicity and identity of representative isolates to fulfill the Koch’s postulates. Seedling leaf inoculation was selected because it is a standardized and widely accepted method for evaluating disease severity and pathogenicity of *M. oryzae* isolates under controlled greenhouse conditions [27,28,29]. Rice plants (cv. Basmati-385) were grown in 2-L earthen pots containing sterilized soil under greenhouse conditions (25 ± 2 °C, 80–90% relative humidity, and a 12-h light/12-h dark photoperiod). One representative isolate from each morphological group (G-I: RBNN-1, G-II: RBSK-3, G-III: RBNR-3, and G-IV: RBGJ-2) was randomly selected for pathogenicity testing. The isolates were selected before pathogenicity evaluation and therefore were not chosen based on disease severity. Inoculum was prepared using the single-spore technique, and conidial suspensions were adjusted to a concentration of 2 × 10^5^ spores mL^−1^ prior to inoculation. At the two- to three-leaf stage, rice seedlings were spray-inoculated with 2–3 mL of conidial suspension per plant using a hand sprayer. For each isolate, a total of 30 plants (10 plants per replication) were inoculated, while control plants were treated with sterile distilled water. Inoculated plants were incubated in a dark dew chamber at 25 °C for 24 h to promote infection and subsequently transferred to the greenhouse. The experiment was arranged in a completely randomized design (CRD) with three replications and was repeated twice.

Disease severity was assessed 10 days after inoculation and subsequently at weekly intervals for four consecutive weeks using the standardized 0–9 disease rating scale of the International Rice Research Institute [30], based on lesion type and affected leaf area. Mean disease severity over four weeks was calculated for each treatment. Disease progression was quantified by calculating the area under the disease progress curve (AUDPC) using the trapezoidal method [31]. Disease severity and AUDPC data were subjected to analysis of variance (ANOVA), and treatment means were separated using the least significant difference (LSD) test at *p* ≤ 0.05. All statistical analysis was performed using R software (version 4.5.2; R Core Team, Vienna, Austria).

Following symptom development, the pathogen was re-isolated from infected tissues and confirmed by comparing morphological characteristics and multilocus sequence data (ITS, β-tubulin, actin, and calmodulin) with the original cultures to fulfill Koch’s postulates.

### 2.4. DNA Extraction, PCR Amplification, and Sequencing

Genomic DNA was extracted from fresh fungal mycelium obtained from actively growing cultures of all 25 representative isolates maintained on PDA and incubated at 26 ± 2 °C for 5–7 days. DNA extraction was performed using the DNeasy Plant Mini Kit (Qiagen, Hilden, Germany) following the manufacturer’s instructions. DNA quality and concentration were assessed using a spectrophotometer (Epoch 2, BioTek, Winooski, VT, USA).

Four genomic regions, including the internal transcribed spacer (ITS1–5.8S–ITS2), β-tubulin, actin and calmodulin genes, were amplified using universal and gene-specific primer pairs. The ITS region was amplified using the universal primers ITS1 and ITS4 [18], while gene-specific primers were used for the remaining loci (Table 1).

PCR products were purified and sequenced bidirectionally (Macrogen Inc., Seoul, Republic of Korea). Resulting sequences were analyzed using the NCBI BLASTn web server (National Center for Biotechnology Information, Bethesda, MD, USA) and deposited in the NCBI GenBank database under the following accession numbers: ITS (MH424730–MH424754), β-tubulin (MH539652–MH539676), Actin (MH898678–MH898680 and MH921396–MH921417) and calmodulin (MH932588–MH932592 and MH936345–MH936364).

### 2.5. Sequence Alignment and Phylogenetic Analysis

Raw sequences were edited and assembled using BioEdit version 7.2.5. Multiple sequence alignments were performed using the MUSCLE algorithm implemented in MEGA version 7.0.26 [33]. Sequences generated in this study were aligned with reference sequences of *Magnaporthe*/*Pyricularia* species retrieved from the GenBank database. Phylogenetic analysis were conducted independently for each gene region. Phylogenetic trees were inferred using the maximum likelihood (ML) method implemented in MEGA with the default nucleotide substitution model. The robustness of the inferred clades was assessed using bootstrap analysis with 1000 replicates. *Cryphonectria parasitica* was used as the outgroup to root all phylogenetic trees.

## 3. Results

### 3.1. Isolation and Morphological Characterization of M. oryzae Isolates

Fungal isolates were successfully obtained from all 125 rice blast-infected leaf and panicle samples collected from Punjab, Pakistan. Based on cultural characteristics, pigmentation, growth pattern, and conidial morphology, the isolates were classified into four distinct morphological groups (G-I–G-IV). From these groups, 25 representative isolates were selected to capture the observed diversity for further analysis. All selected isolates were purified and maintained on PDA, and both cultural and microscopic examinations confirmed their identity as *M. oryzae*.

**Figure 2:**
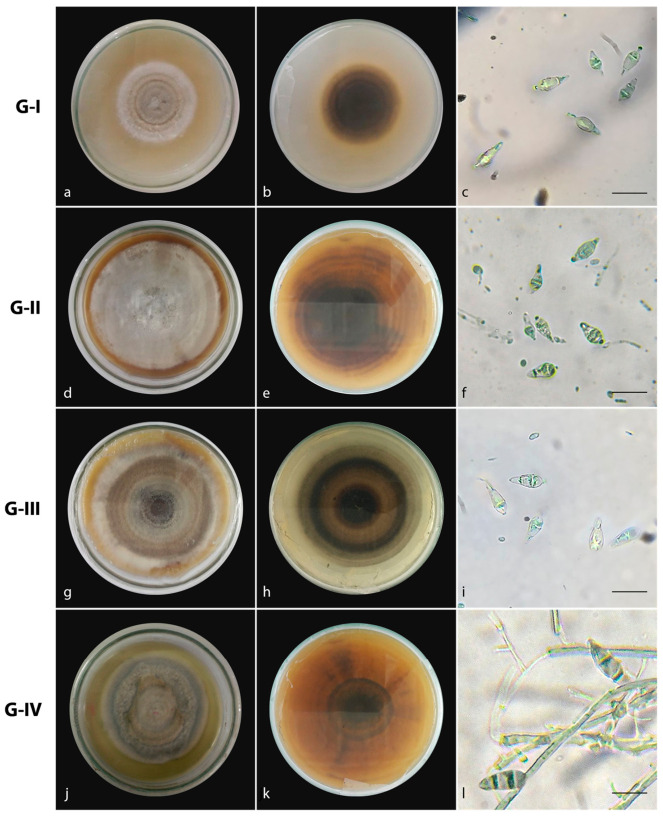
Colony morphology and conidial characteristics of *M. oryzae* isolates grouped into four morphological types. (**a**–**c**) Group I; (**d**–**f**) Group II; (**g**–**i**) Group III; (**j**–**l**) Group IV, showing colony appearance from both sides and corresponding conidial morphology. Conidia are hyaline, pyriform, and biseptate. Scale bars = 20 µm.

Group I comprised seven isolates (RBNN-1, RBNN-4, RBNN-5, RBNR-1, RBNR-4, RBHF-4, and RBHF-5) characterized by white colonies with ring-like, smooth margins. The conidia were small, pyriform, measuring approximately 20.6 × 7.5 μm, typically with two septa, and were produced on hyaline, septate hyphae. Group II included seven isolates (RBNN-2, RBNN-3, RBSK-2, RBSK-3, RBSK-4, RBSK-5, and RBGJ-1) showing white colonies with smooth circular margins. These isolates produced medium-sized, pyriform conidia (21.4 × 8.1 μm) with two septa, along with hyaline, septate hyphae. Group III consisted of five isolates (RBNR-2, RBNR-3, RBNR-5, RBSK-1, and RBGJ-5) exhibiting grayish-white colonies with smooth circular margins and raised mycelium. The conidia were small (20.4 × 7.7 μm), pyriform, and biseptate, while the hyphae were hyaline and septate. Group IV comprised six isolates (RBHF-1, RBHF-2, RBHF-3, RBGJ-2, RBGJ-3, and RBGJ-4) characterized by creamy white colonies with ring-like, cottony margins. These isolates produced comparatively larger pyriform conidia (22.8 × 8.1 μm) with two septa and hyaline, septate hyphae (Figure 2).

### 3.2. Pathogenic Variability Among M. oryzae Isolates

**Figure 3:**
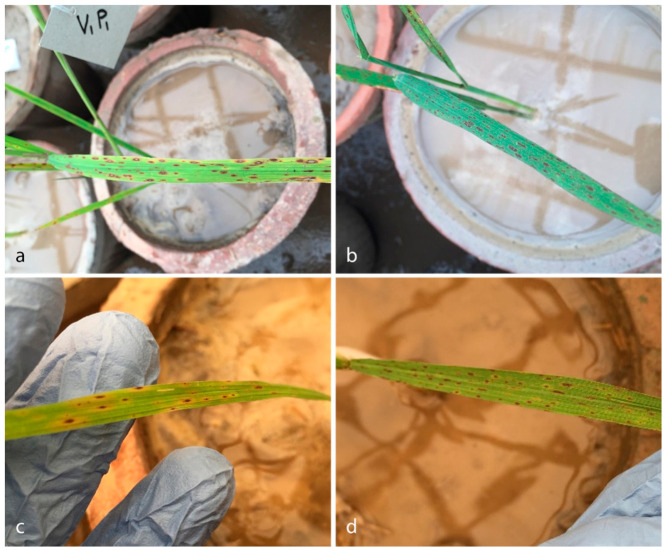
Pathogenicity of *M. oryzae* isolates on rice leaves under greenhouse conditions. Photographs were captured at the final disease assessment (Week 4 of the disease evaluation period). Typical rice blast symptoms are shown on plants inoculated with (**a**) RBNN-1, (**b**) RBGJ-2, (**c**) RBSK-3, and (**d**) RBNR-3, characterized by spindle-shaped lesions with necrotic centers.

All four tested *M. oryzae* isolates successfully induced typical rice blast symptoms on inoculated plants under greenhouse conditions, whereas no symptoms were observed in the non-inoculated control, confirming their pathogenic nature (Figure 3).

Significant differences (*p* ≤ 0.05) in mean disease severity were observed among the isolates. Mean disease severity after four weeks ranged from 18.93% to 32.70%. The isolate RBNN-1 exhibited the highest mean disease severity (32.70%), followed by RBGJ-2 (24.68%), RBSK-3 (21.40%), and RBNR-3 (18.93%). All isolates differed significantly from each other, as indicated by distinct letter groupings (Table 2; Supplementary Table S2).

Disease progression, expressed as area under the disease progress curve (AUDPC), also varied significantly among isolates (*p* ≤ 0.05). The highest AUDPC value was recorded for RBNN-1 (97.75), indicating greater disease development over time, followed by RBGJ-2 (73.80), RBSK-3 (64.20), and RBNR-3 (56.75). The control treatment showed no disease development (AUDPC = 0.00). Differences among isolates were statistically significant, as reflected by the separation of means into distinct groups.

Overall, the results demonstrated clear variation in disease development among *M. oryzae* isolates. RBNN-1 produced the highest disease severity and AUDPC values under the tested greenhouse conditions, whereas RBNR-3 exhibited the lowest disease severity and AUDPC among the inoculated isolates.

### 3.3. PCR Amplification of Target Gene Regions

**Figure 4:**
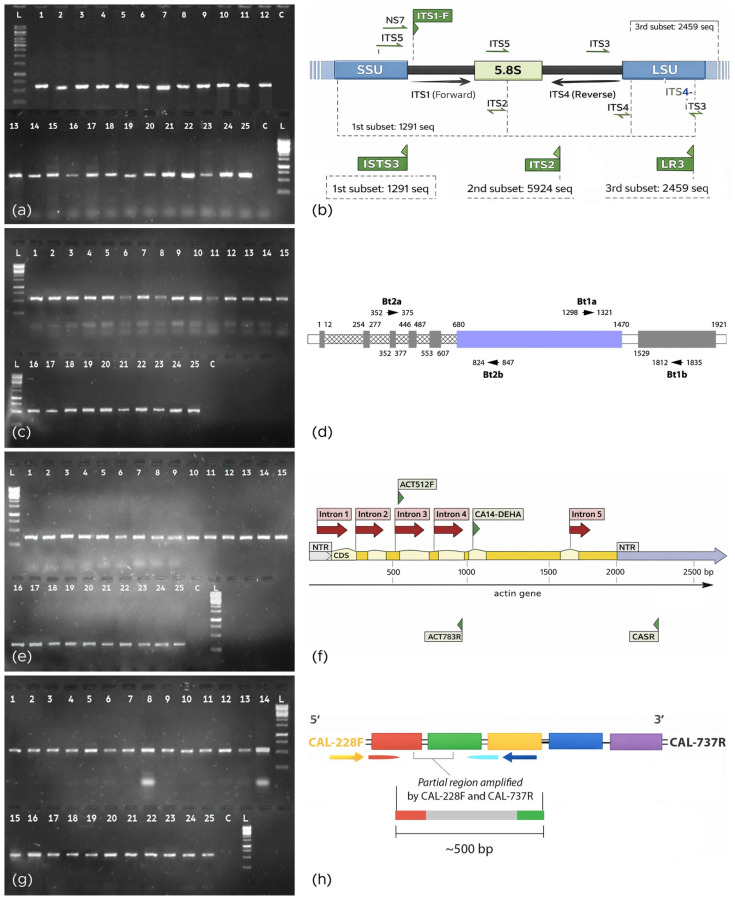
PCR amplification of *M. oryzae* isolates using universal and gene-specific primers. (**a**) Gel electrophoresis showing amplification of the ITS region with expected amplicon size of ~510 bp, with its corresponding schematic representation in (**b**). (**c**) Amplification of the β-tubulin gene showing ~500 bp fragments, with the amplified region illustrated in (**d**). (**e**) Amplification of the actin gene producing ~280 bp products, with the corresponding gene structure shown in (**f**). (**g**) Amplification of the calmodulin gene yielding ~520 bp amplicons, with the amplified region indicated in (**h**). L denotes the 1 kb DNA ladder and C represents the negative control.

PCR amplification was successfully achieved for all four target genomic regions across the 25 *M. oryzae* isolates. The ITS region produced a clear amplicon of approximately 510 bp, while the β-tubulin gene yielded an amplicon of approximately 500 bp. The actin gene generated a fragment of approximately 280 bp, and the calmodulin gene produced an amplicon of approximately 520 bp (Figure 4).

### 3.4. Molecular Characterization and Sequence Analysis

Bidirectional sequencing of all amplified products confirmed that all isolates belonged to *M. oryzae*, showing ≥99% sequence similarity with reference sequences retrieved from the NCBI GenBank database, including representative accessions from the ITS region (e.g., LK932245), β-tubulin (e.g., JX014265), actin (e.g., DQ240884), and calmodulin (e.g., KM485261). The obtained sequences were of high quality and suitable for downstream alignment and phylogenetic analysis. Sequence alignment revealed limited genetic variation among the analyzed loci. Among the four genomic regions, variation was detected only in the ITS region. A single nucleotide substitution (A→G) was identified at position 413 bp in seven isolates (RBNN-1, RBNN-4, RBNN-5, RBNR-1, RBNR-4, RBHF-4, and RBHF-5) (Supplementary Figure S1). These isolates belonged to morphological Group I and were associated with higher disease severity under greenhouse conditions.

### 3.5. Phylogenetic Analysis and Genetic Structure of M. oryzae Populations

**Figure 5:**
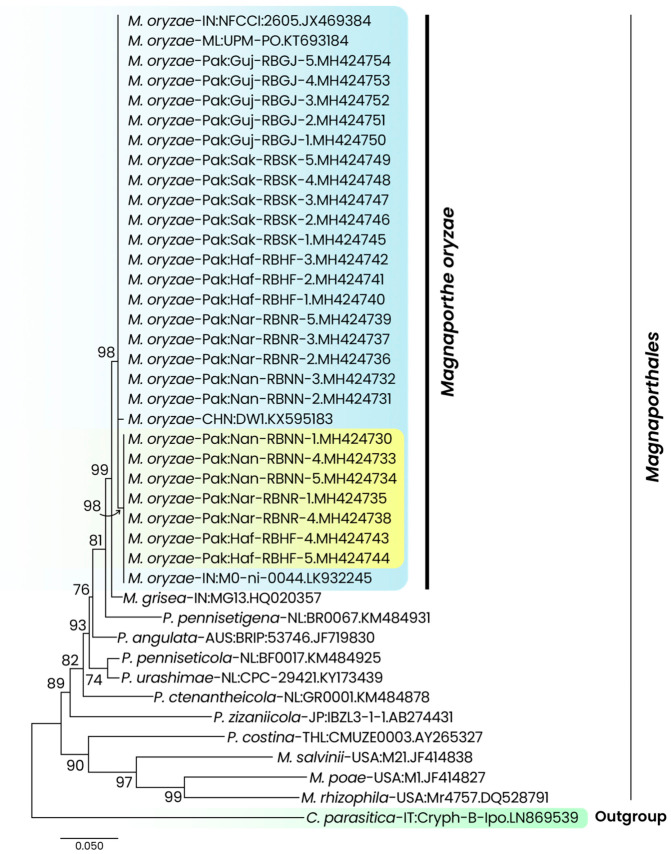
Maximum likelihood phylogenetic tree based on ITS sequences showing relationships among *M. oryzae* isolates. All isolates clustered within a single well-supported clade, with a distinct subclade (bootstrap = 98%) representing isolates carrying a single nucleotide polymorphism (A→G) in the ITS region. Bootstrap values are indicated at the nodes. The scale bar represents the number of nucleotide substitutions per site.

**Figure 6:**
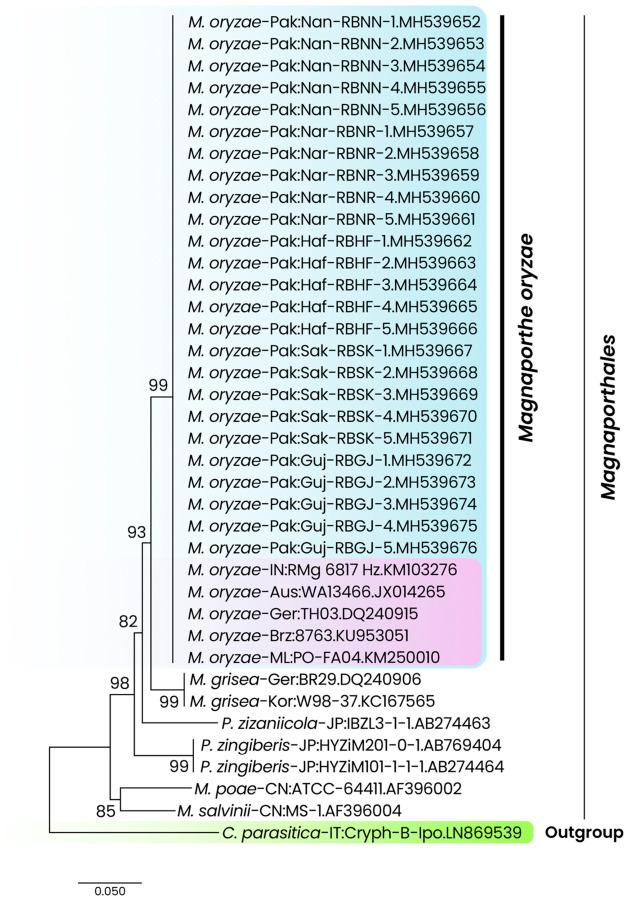
Maximum likelihood phylogenetic tree based on β-tubulin gene sequences showing relationships among *M. oryzae* isolates. All isolates clustered within a single well-supported clade with high bootstrap support, indicating high genetic uniformity. Bootstrap values are shown at the nodes. The scale bar represents the number of nucleotide substitutions per site, and *C. parasitica* was used as an outgroup.

**Figure 7:**
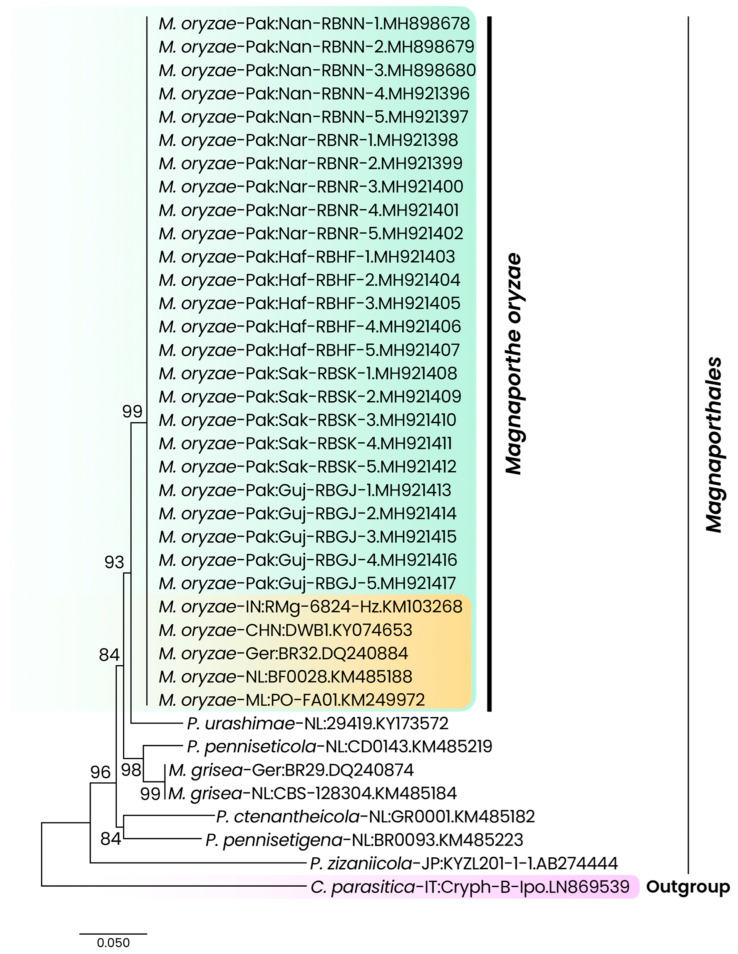
Maximum likelihood phylogenetic tree constructed from actin gene sequences illustrating the genetic relationships among *M. oryzae* isolates. All isolates grouped within a single highly supported clade, reflecting a high level of sequence conservation.

**Figure 8:**
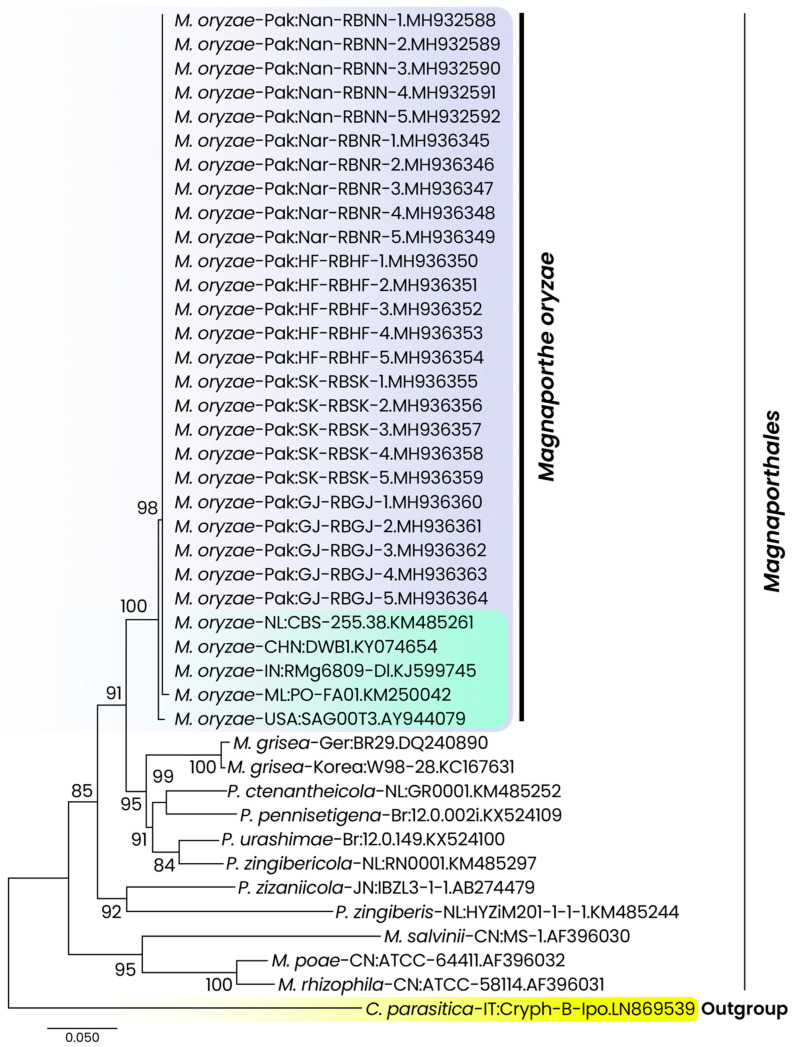
Maximum likelihood phylogenetic tree inferred from calmodulin gene sequences showing the genetic relationships among *M. oryzae* isolates. All isolates clustered within a single highly supported clade with strong bootstrap values, indicating a high level of sequence conservation. Bootstrap values are displayed at the nodes.

Phylogenetic relationships among the 25 *M. oryzae* isolates were inferred independently for four genomic regions (ITS, β-tubulin, actin and calmodulin) using maximum likelihood analysis. Across all loci, the Pakistani isolates consistently clustered within a well-defined *M. oryzae* clade, clearly separated from the outgroup, confirming species identity. The ITS-based phylogenetic tree revealed limited ITS sequence variation among the isolates. Although all isolates clustered within a single major *M. oryzae* clade supported by high bootstrap values (≈98–99%), seven isolates carrying an ITS-defined haplotype formed a distinct and well-supported subclade (bootstrap = 98%) (Figure 5). These isolates belonged to morphological Group I and consistently exhibited higher disease severity and AUDPC under the tested greenhouse conditions. In contrast, phylogenetic analysis based on β-tubulin, actin, and calmodulin gene sequences demonstrated a high level of genetic uniformity among the Pakistani isolates. In the β-tubulin tree, all isolates clustered into a single clade with strong bootstrap support (≈99%), with no clear internal subdivision (Figure 6). Similarly, actin- and calmodulin-based phylogenies showed all isolates grouped into a highly supported monophyletic clade (bootstrap values ranging from 98% to 100%), indicating a high degree of sequence conservation across these protein-coding loci (Figure 7 and Figure 8).

Overall, multilocus sequence analysis demonstrated a high degree of sequence conservation across the β-tubulin, actin and calmodulin loci among the analyzed isolates. In contrast, limited but distinct ITS sequence variation was observed within the ITS region. The isolates carrying the ITS-defined haplotype formed a well-supported subclade and were consistently associated with a distinct morphological group and higher disease severity among the analyzed isolates.

## 4. Discussion

In Pakistan, rice blast diagnosis has traditionally relied on field symptoms and morphological characteristics of the pathogen. Although these approaches are useful for preliminary identification, they are insufficient to resolve intraspecific diversity or explain variation in pathogenic behavior [18]. In the present study, an integrated approach combining morphological characterization, pathogenicity assays and multilocus molecular analysis provided new insights into the characterization of *M. oryzae* isolates collected from Punjab, Pakistan. Unlike many previous studies that have primarily focused on either molecular diversity or host specificity, this study integrates morphological characterization, pathogenicity assays and multilocus molecular analysis to provide a more comprehensive characterization of *M. oryzae* isolates from Punjab, Pakistan.

Morphological characterization revealed clear phenotypic diversity among the analyzed isolates, which were grouped into four distinct categories based on cultural characteristics and conidial morphology. Although morphological traits may be influenced by environmental conditions and have limited taxonomic resolution, they remain valuable for the preliminary characterization of *M. oryzae* isolates when integrated with molecular analysis. Similar integrative approaches combining morphological observations with multilocus molecular characterization have been widely used for the identification and characterization of *M. oryzae* and related *Pyricularia* species [23,34,35]. In the present study, the morphological grouping served as a practical framework for selecting representative isolates for subsequent pathogenicity and molecular analysis.

The pathogenicity assays revealed significant differences in disease severity and AUDPC among the tested isolates. The isolate RBNN-1 consistently produced the highest disease severity among the tested isolates. While previous studies have documented genetic diversity and host specialization in *M. oryzae* populations [13,23], relatively few studies have integrated morphological characterization, pathogenicity assays, and multilocus molecular analysis to comprehensively characterize representative isolates within a single investigation. The present study contributes to addressing this gap by demonstrating that representative isolates can exhibit significant differences in disease severity and AUDPC even within a limited morphological and genetic spectrum. These findings highlight the importance of integrating phenotypic assays with molecular characterization when comparing representative isolates. The observed differences in disease severity and AUDPC may also be influenced by variation in virulence-associated genes, particularly avirulence (AVR) effectors, which play important roles in host recognition and disease development in *M. oryzae* [36,37]. Although effector profiling was beyond the scope of the present study, future characterization of major AVR genes, including *AvrPita*, *AvrPik*, *AvrPi9*, and *AvrPib*, would provide valuable insights into the genetic basis of the pathogenic variation observed among these isolates. A limitation of the present study is that pathogenicity was evaluated using a standardized seedling leaf inoculation assay. Consequently, tissue-specific specialization, such as neck or panicle blast, was not assessed and should be investigated in future studies.

The multilocus markers employed in this study were selected because they are widely used for species identification and phylogenetic characterization of *M. oryzae* and related *Pyricularia* species rather than for fine-scale population genetic or race differentiation analysis [21,22,25]. The protein-coding genes β-tubulin, actin, and calmodulin were highly conserved across isolates, consistent with their essential roles and slower evolutionary rates [17,18]. In contrast, the ITS region exhibited limited sequence variation, with a single nucleotide polymorphism consistently distinguishing a subset of isolates. Although the ITS region is primarily used for species identification and phylogenetic characterization, previous studies have shown that in some fungal taxa it may reveal intraspecific sequence variation, although its discriminatory power varies among species [18,38,39].

Phylogenetic analysis identified an ITS-defined haplotype, with the corresponding isolates forming a well-supported subclade. Importantly, these isolates were consistently associated with a distinct morphological group and higher disease severity under the tested greenhouse conditions. Although the detected variation was limited to a single nucleotide substitution, this ITS-defined haplotype was consistently identified by bidirectional sequencing and pathogen re-isolation following Koch’s postulates and was associated with a distinct morphological group and higher disease severity among the analyzed isolates. The detection of a similar ITS sequence variant in an independent GenBank accession from India (Accession No. LK932245) suggests that this ITS-defined haplotype may not be unique to the isolates analyzed in the present study. Although additional sampling is required to determine its geographic distribution and frequency, this finding suggests that similar ITS sequence variants may occur in *M. oryzae* populations from other rice-growing regions. The present findings also highlight the importance of selecting molecular markers according to the objectives of the study. The loci employed in this study were selected for species identification and multilocus phylogenetic characterization rather than comprehensive population genetic analysis. While the protein-coding loci were highly conserved, the ITS region identified a reproducible ITS-defined haplotype associated with a distinct morphological group and higher disease severity among the analyzed isolates. Future studies employing higher-resolution approaches, including SSR markers, genome-wide SNP analysis, whole-genome sequencing, and population genetic analysis such as STRUCTURE and principal component analysis (PCA), will further improve the understanding of population structure, evolutionary relationships and the biological significance of this ITS-defined haplotype.

Overall, this study represents the first comprehensive characterization of *M. oryzae* isolates from the major rice-growing regions of Punjab, Pakistan, integrating morphological characterization, quantitative pathogenicity assessment and multilocus phylogenetic analysis. Although the protein-coding loci were highly conserved, the ITS-defined haplotype identified in this study was consistently associated with a distinct morphological group and differences in disease severity among the analyzed isolates. These findings provide a valuable baseline for future investigations of *M. oryzae* diversity in Pakistan and establish a foundation for high-resolution genomic and population genetic studies aimed at improving our understanding of pathogen evolution, host–pathogen interactions and the development of sustainable rice blast management strategies.

## Figures and Tables

**Table 1:** Primer pairs, sequences and expected amplicon sizes used for PCR amplification and sequencing of *M. oryzae*.

Target Genes	Primer	Primer DNA Sequence (5′–3′)	Amplicon Size (bp)	Reference
ITS	ITS1-F ITS4-R	TCCGTAGGTGAACCTGCGG TCCTCCGCTTATTGATATGC	500–550	[20]
β-tubulin	Bt1a-F Bt1b-R	TTCCCCCGTCTCCACTTCTTCATG GACGAGATCGTTCATGTTGAACTC	500–550	[32]
Actin	ACT-512F ACT-783R	ATGTGCAAGGCCGGTTTCGC TACGAGTCCTTCTGGCCCAT	200–300	[19]
Calmodulin	CAL-228F CAL-737R	GAGTTCAAGGAGGCCTTCTCCC CATCTTTCTGGCCATCATGG	500–550	[19]

PCR reactions were carried out in a 25-μL volume using DreamTaq Green Master Mix under the following cycling conditions: initial denaturation at 94 °C for 2 min; followed by 35 cycles of denaturation at 94 °C for 30 s, annealing at 52–55 °C for 30 s, and extension at 72 °C for 1 min; with a final extension at 72 °C for 10 min. Amplified products were resolved on 1% agarose gels and visualized under UV illumination. A negative (no-template) control was included in each PCR run to monitor contamination. A reference *M. oryzae* strain was not available for use as a positive control. Therefore, species identity was confirmed by bidirectional sequencing, BLAST analysis and multilocus phylogenetic analysis of all amplified products.

**Table 2:** Effect of *M. oryzae* isolates on disease severity (%) at 4 weeks and AUDPC of rice under greenhouse conditions. Values represent mean disease severity (%) at 4 weeks after inoculation and AUDPC ± SE (n = 30 plants per treatment; 10 plants per replication). Means followed by different letters within a column are significantly different according to the LSD test at *p* ≤ 0.05.

Isolate	Mean Severity (%) ± SE	AUDPC ± SE
RBNN-1	32.70 ± 1.20 a	97.75 ± 3.15 a
RBGJ-2	24.68 ± 0.95 b	73.80 ± 2.10 b
RBSK-3	21.40 ± 0.80 c	64.20 ± 1.85 c
RBNR-3	18.93 ± 0.85 d	56.75 ± 1.92 d
Control	0.00 ± 0.00 e	0.00 ± 0.00 e

## Data Availability

The data presented in this study are available within the article and its Supplementary Materials. Sequence data have been deposited in the NCBI GenBank database under the accession numbers provided in the manuscript.

## Supplementary Materials

The following supporting information can be downloaded at: https://www.mdpi.com/article/10.3390/jof12070501/s1, Table S1. Sampling information for rice blast-infected samples collected from Punjab, Pakistan, including sampling districts, villages, village GPS coordinates, number of fields sampled, host (*Oryza sativa*) and plant tissues collected during the 2016–2018 cropping seasons; Table S2. Weekly disease severity (%) of *M. oryzae* isolates under greenhouse conditions; Figure S1. Multiple sequence alignment of the ITS region of *M. oryzae* isolates showing a single nucleotide substitution (A→G) at position 413 bp in selected isolates. All sequences showed ≥99% similarity with reference sequences from the NCBI GenBank database, confirming species identity.
