# Supplementary material for: Multilocus Characterization Reveals an ITS-Defined Haplotype Associated with Pathogenic Variation in Magnaporthe oryzae
Source: J Fungi (Basel). 2026 Jul 9;12(7):501. doi: 10.3390/jof12070501 (PMC13412558; doi:10.3390/jof12070501)
Supplement: Supplementary file 1 [file jof-12-00501-s001.zip › jof-4350860-supplementary.pdf]

Supplementary Data:

Table S1: Sampling information for rice blast-infected samples collected from Punjab, Pakistan, including sampling districts, villages, village GPS coordinates, number of fields sampled, host (*Oryza sativa*) and plant tissues collected during the 2016-2018 cropping seasons.

Districts	Villages	Number of fields sampled	Coordinates	Host	Plant portion
Gujranwala	Korotana	5	32°04'47.9"N 74°10'31.1"E	*O. sativa*	Leaves
	Gagewali	5	32°14'33.9"N 74°15'00.8"E	*O. sativa*	Leaves
	Verpal Chatha	5	32°15'29.0"N 73°54'03.4"E	*O. sativa*	Leaves
	Nowshera Virkan	5	31°57'19.7"N 73°58'07.4"E	*O. sativa*	Leaves
	Pir Kot	5	32°18'44.3"N 74°06'57.8"E	*O. sativa*	Leaves/panicles
Nankana	Chak 9 GB	5	31°29'36.0"N 73°33'08.6"E	*O. sativa*	Leaves/panicles
	Safdarabad	5	31°37'35.5"N 73°45'10.3"E	*O. sativa*	Leaves
	Morar Kalaan	5	31°42'13.9"N 73°24'13.6"E	*O. sativa*	Leaves/panicles
	Kot Hira Das	5	31°26'18.0"N 73°43'04.8"E	*O. sativa*	Leaves
	More Khunda	5	31°19'19.1"N 73°48'11.7"E	*O. sativa*	Leaves
Hafizabad	Bugga	5	32°02'05.4"N 73°49'09.0"E	*O. sativa*	Leaves/panicles
	Jalalpur Bhattian	5	32°03'53.3"N 73°22'12.1"E	*O. sativa*	Leaves
	Ramke Chattha	5	32°12'54.3"N 73°39'45.2"E	*O. sativa*	Leaves/panicles
	Pindi Bhattian	5	31°54'11.9"N 73°18'00.4"E	*O. sativa*	Leaves
	Hanjrawan Wala	5	31°57'40.1"N 73°41'59.4"E	*O. sativa*	Leaves

Sialkot	Seoki	5	32°20'52.1"N 74°28'05.3"E	*O. sativa*	Leaves
	Sambrial	5	32°28'07.8"N 74°20'53.5"E	*O. sativa*	Leaves
	Kotli Loharan	5	32°34'56.3"N 74°29'38.4"E	*O. sativa*	Leaves
	Dheera Sandha	5	32°27'10.7"N 74°34'16.2"E	*O. sativa*	Leaves
	Chobara	5	32°24'11.3"N 74°47'28.0"E	*O. sativa*	Leaves
Narowal	Jassar	5	32°05'44.2"N 74°56'28.3"E	*O. sativa*	Leaves
	Baddomalhi	5	31°59'44.1"N 74°40'21.3"E	*O. sativa*	Leaves
	Darman	5	32°24'33.0"N 74°59'50.1"E	*O. sativa*	Leaves
	B. Chada Kalan	5	32°14'27.6"N 74°48'39.7"E	*O. sativa*	Leaves/panicles
	Jarpal	5	32°09'31.3"N 75°09'30.1"E	*O. sativa*	Leaves

Table S2. Weekly disease severity (%) of *M. oryzae* isolates under greenhouse conditions.

Isolate	Week 1	Week 2	Week 3	Week 4
RBNN-1	21.8	28.5	36.2	44.3
RBGJ-2	18.9	23.2	26.5	30.8
RBSK-3	17.2	19.8	23.4	25.6
RBNR-3	14.5	17.1	20.6	23.4
Control	0.0	0.0	0.0	0.0

Values represent mean disease severity (%) recorded at weekly intervals after inoculation (n = 30 plants per treatment; 10 plants per replication).

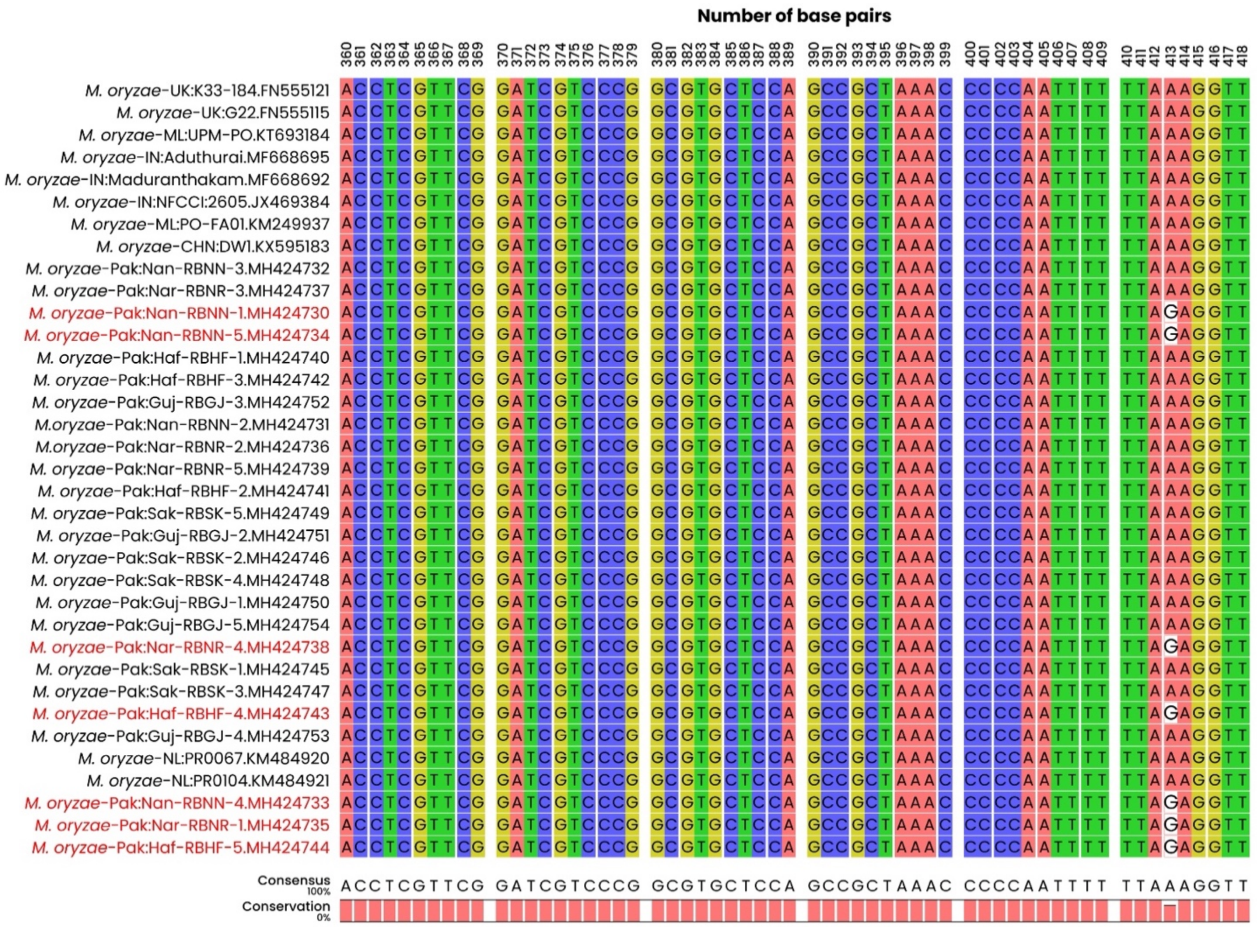

Figure S1: Multiple sequence alignment of the ITS region of *M. oryzae* isolates showing a single nucleotide substitution (A→G) at position 413 bp in selected isolates. All sequences showed ≥99% similarity with reference sequences from the NCBI GenBank database, confirming species identity.
